# Rapid Urbanization of Red Foxes in Estonia: Distribution, Behaviour, Attacks on Domestic Animals, and Health-Risks Related to Zoonotic Diseases

**DOI:** 10.1371/journal.pone.0115124

**Published:** 2014-12-22

**Authors:** Liivi Plumer, John Davison, Urmas Saarma

**Affiliations:** Department of Zoology, Institute of Ecology and Earth Sciences, University of Tartu, Tartu, Estonia; University of Tasmania, Australia

## Abstract

Urban areas are becoming increasingly important for wildlife as diminishing natural habitats no longer represent a suitable environment for many species. Red foxes (*Vulpes vulpes*) are nowadays common in many cities worldwide, and in recent years they have colonized urban areas in Estonia. We used a public web-based questionnaire approach to evaluate the distribution and behaviour of Estonian urban foxes, to detect related problems and to assess health risks to humans and domestic animals. In total, 1205 responses were collected throughout the country. Foxes have colonized the majority of Estonian towns (33 out of 47) in a relatively short period of time, and have already established breeding dens in several towns. Despite their recent arrival, the behaviour of Estonian urban foxes is similar to that reported in longer-established urban fox populations: they are mostly active during night-time, often visit city centres and some also have dens in such locations. Certain characteristics of urban foxes serve as a basis for conflict with humans: foxes have entered houses and attacked domestic animals, killing cats and poultry. About 8% of reported foxes exhibited symptoms of sarcoptic mange, a disease that also infects domestic animals, especially dogs. The proportion of mange-infected foxes was higher in large urban areas. In addition to mange, a substantial fraction of red foxes in Estonia are known to be infected with the life-threatening tapeworm *Echinococcus multilocularis*, the causative agent of alveolar echinococcosis. Therefore, urban foxes may represent a source of serious infectious disease for pets and humans.

## Introduction

Expanding cities and the general trend towards urbanization are changing natural environments worldwide [1]. As natural areas give way to highly modified landscapes, the habitats used by wildlife can be lost or fragmented, leading to declines in biodiversity [2]. The questions of how wildlife adapts to urbanization and what issues accompany urban wildlife are becoming increasingly important as the global human population continues to grow [3]. Even in cities where both population and household numbers have declined, the urban land area has nevertheless continued to increase due to an increase in the per capita living space [4]. These trends, coupled with an increasing number of domesticated animals in urban areas, mean that the potential for hybridization between wild and closely related domestic animals is increasing, which is especially relevant for canids (e.g. [5], [6]).

Some organisms are capable of exploiting urban environments better than others, and among mammals, medium body size, social behaviour and behavioural flexibility, including diet, appear to be the main factors associated with successful urbanization. Consequently, species such as red fox (*Vulpes vulpes*), coyote (*Canis latrans*), raccoon (*Procyon lotor*) and badger (*Meles meles*) can be found in cities around the world [7], though smaller mammals including bats, squirrels, hedgehogs, mice and voles, are also widespread in urban areas (e.g. [3], [8]). Some urban mammals even establish synurbic populations living at far higher density than nearby rural populations [9], such as red foxes in Switzerland [10].

Red foxes were among the first urban mammals to be subjected to scientific investigation. When they started to occur in British cities in the 1930s, it appeared to represent a local phenomenon, restricted to Great Britain [11]. However, similar colonization events took place in many Central-European cities in the 1980s, following successful oral vaccination campaigns against rabies in wildlife [12]. It appeared therefore that red foxes colonized urban areas during periods of sudden population growth.

There has been considerable interest in understanding the behaviour of urban foxes. Thorough investigations have been made of foxes living in Bristol, Great Britain [13], [14], [15], Zurich, Switzerland [16], [17], [10] and Grünwald, Germany [18], [19]. Meanwhile, Gloor et al. [10] investigated foxes in multiple towns in the German-speaking part of Switzerland. These studies have revealed a number of behavioural differences between urban and rural foxes. For instance, Baker et al. [20] found that urban foxes changed their activity patterns to reduce the risk of mortality presented by traffic collisions. Foxes in urban areas also tend to rely on anthropogenic food items [16] and consume fewer rodents compared to animals in natural environments [17]. As a consequence of resource patterns, the home-range sizes of foxes also tend to be smaller in urban than in rural areas [18].

A major source of conflict between humans and urban foxes is the disease-risk that foxes can pose for domestic animals and humans. Foxes can spread infectious parasitic and viral diseases that are highly dangerous to humans and/or domestic animals, e.g. alveolar echinococcosis [12], rabies [21] and sarcoptic mange [22]. Alveolar echinococcosis, caused by the parasitic tapeworm *Echinococcus multilocularis*, is a severe disease with a high mortality rate in humans [23]. Humans can become infected with parasite eggs through direct contact with fox feces, which could occur through the intermediary of domestic dogs, if their coats or muzzles become contaminated. The mite that causes sarcoptic mange, *Sarcoptes scabiei var. vulpes*, infects dogs and cats as well as foxes [24].

There are 47 towns in Estonia with 1,000–400,000 human inhabitants. Red foxes started to colonize urban areas in Estonia shortly after the initiation of a successful anti-rabies campaign in wild animals in 2005 [21]. The urbanization of red foxes has attracted considerable public attention in Estonia, especially since 2008, when their occurrence in urban areas appeared to increase sharply. News items on urban foxes and their behaviour have become common in the public media, and the general public has expressed various attitudes towards foxes, ranging from appreciation to serious concern.

Surveys engaging local residents represent one of the most efficient ways to gather information about urban foxes. Sadlier et al. [25] noted that questionnaires or sighting surveys are more suitable in urban than rural areas, where direct fox observation is relatively rare. Nevertheless, such studies are still relatively scarce. Questionnaire surveys directed to the public and wildlife managers have been used effectively to collect information on urban fox abundance and distribution [3], [10], [26], the incidence of sarcoptic mange [14], public knowledge about *E. multilocularis* [27] and attitudes towards urban foxes [28]; and public data sets have been used to estimate the number of families in urban fox populations [29]. However, questionnaire surveys are potentially prone to problems such as reporting error and standardization of effort [25]. People with established opinions are more willing to respond to surveys than people who have neutral or indistinct views. To investigate the distribution and behaviour of red foxes in Estonian towns we invited members of the public throughout Estonia to complete a web-based questionnaire about foxes seen in their hometowns. This was designed to determine: (i) how many towns in Estonia have been colonized by red foxes, (ii) what behaviours urban foxes display, and (iii) what proportion of urban foxes are infected with sarcoptic mange. Our main motivation for investigating the distribution and behaviour was to assess potential health risks for humans and domestic animals. As urban foxes may transmit zoonotic diseases it is important to inform the public about potential risks associated with foxes.

## Materials and Methods

We set up a web-based questionnaire (S1 Information) directed at people who had seen foxes in an Estonian town. The questions sought to find out how many Estonian towns are regularly visited by red foxes, what types of activity the foxes exhibit and whether they are infected with sarcoptic mange. The questionnaire was widely advertised in Estonian national newspapers and radio broadcasts in January 2011. It consisted of 26 questions that fell into two groups: (i) the time, location and conditions of a fox observation (18 questions); and (ii) additional questions to people who observed a fox in the vicinity of their home (8 questions). Some questions were formulated as yes/no choices, some were open questions and some presented relevant information to be ticked. The questionnaire was open from January 2011 till September 2012. Ethical approval was not required since the questionnaire was anonymous and the authors had no access to any identifying information. Participants were notified before responding that their answers would be used for research purposes. Relationships between numbers of records and categorical factors were analysed using χ^2^ tests. Generalized linear models (GLM; R Core team 2014) with binomial error structures were used to determine whether (i) the proportion of records occurring in winter (December, January and February) was related to the size of the urban area; and (ii) the proportion of records reporting foxes with mange was related to the size of the urban area. We used two definitions of urban area size: the area (km^2^), and the human population size in 2012.

## Results

In total, 1205 responses were collected (S2 Information). Of these, 1047 (87%) were first time responses and the remaining 158 (13%) were from participants who saw foxes more than once. 57% of answers came from women and 43% from men. Respondents were most commonly between 21–30 and 31–40 years of age (S1 Figure).

### Foxes have colonized most towns in Estonia

**Figure 1:**
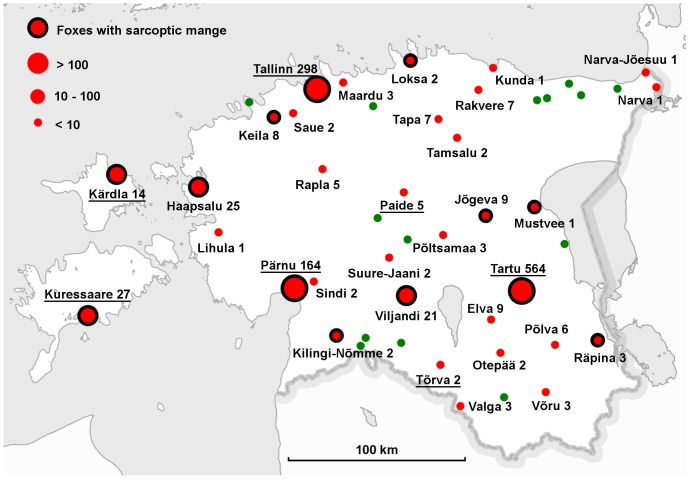
Red foxes in urban areas in Estonia. Foxes were reported in 33 towns (red points) out of 47. The numbers on the map indicate the numbers of people reporting fox sightings. Black rings around red points indicates towns where foxes with symptoms of sarcoptic mange were reported. Green points indicate the 14 towns where urban foxes were not reported. Underlining indicates towns where fox dens were reported.

**Figure 2:**
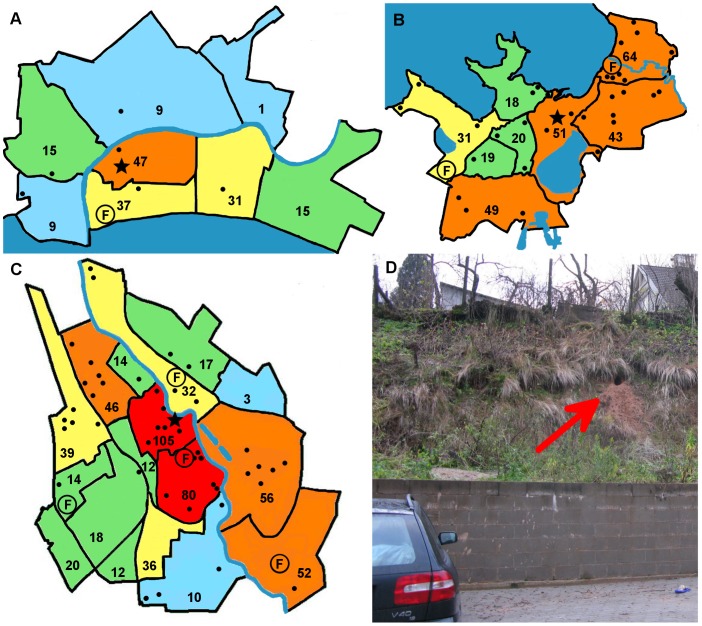
Distribution of foxes in the three largest cities in Estonia: Pärnu (A), Tallinn (B) and Tartu (C), and a photo of a fox den in the central part of Tartu (D, indicated by arrow). The city centre is denoted by a black star, and numbers indicate the number of people reporting fox sightings in different parts of the city. The black points indicate reports of foxes exhibiting symptoms of sarcoptic mange. Fox dens are shown by the letter F.

Foxes were reported in 33 out of 47 Estonian towns (Fig. 1). Most responses came from the three largest towns in the country: Tartu (47% of all responses, 564), Tallinn (the capital of Estonia, 25%, 298) and Pärnu (14%, 164). People were also asked to specify the district in each town, and it became evident that foxes were seen most often in the central parts of these towns (Fig. 2). The number of observations was correlated with the size of the cities: foxes were more frequently reported in larger (km^2^; r = 0.53; p<0.01) and more populated towns (r = 0.62; p<0.01). Fox dens were reported in six towns: four in Tartu, two each in Tallinn and Kuressaare, and one each in Tõrva, Kärdla and Pärnu (Fig. 1).

**Figure 3:**
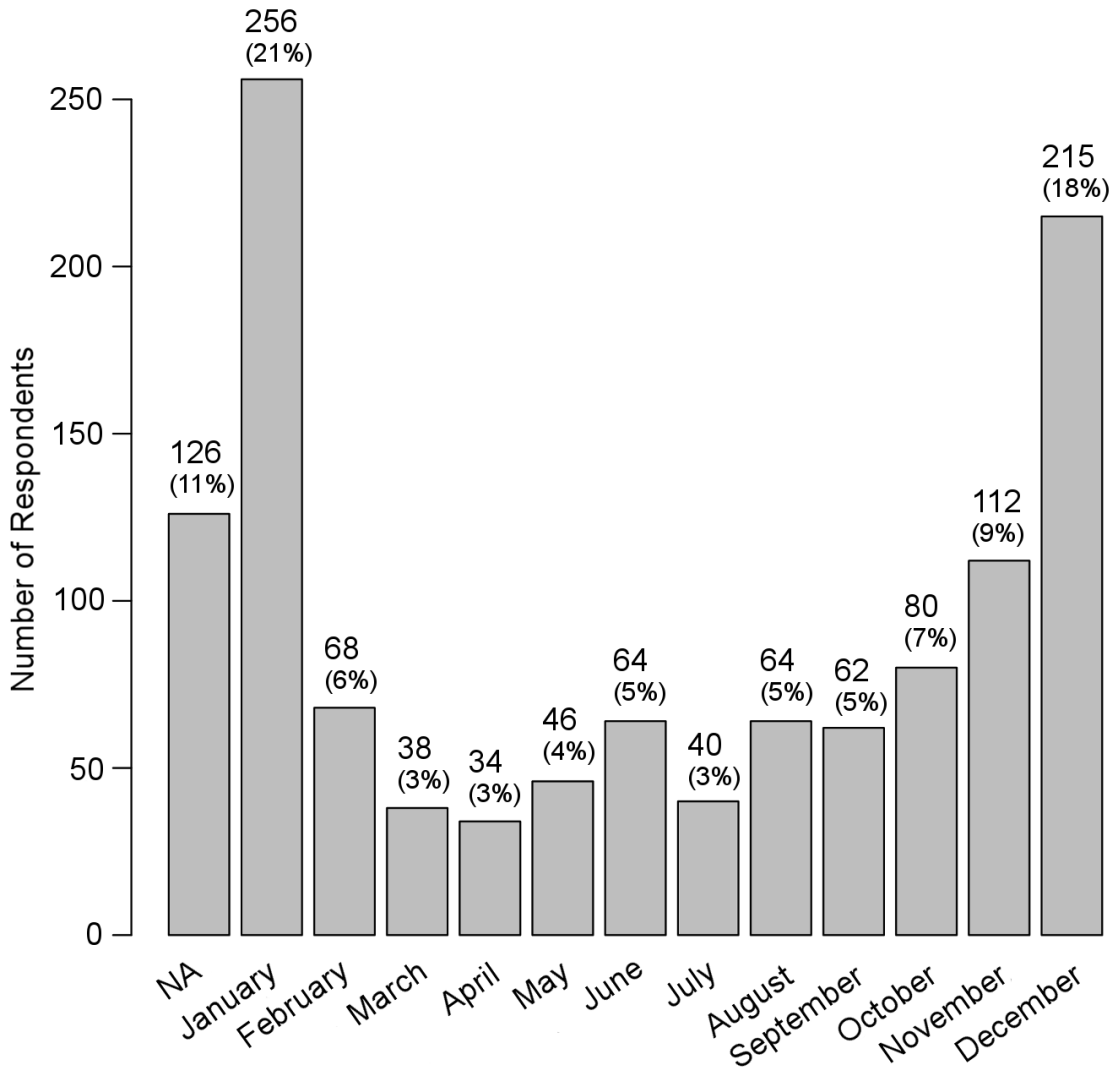
Distribution of fox sightings in different months. NA denotes sightings where the month was not specified.

Foxes were most frequently sighted in winter, particularly in December and January (Fig. 3). The proportion of records occurring in winter (December, January and February) was generally higher in larger towns though the relationship was marginally non-significant (area, GLM; χ^2^ = 2.66; df = 1; p = 0.10; parameter estimate ±s.e. = 0.0018±0.0011) (population size, GLM; χ^2^ = 1.73; df = 1; p = 0.18; parameter estimate ±s.e. = 5.5e^−7^±4.2e^−7^). In about half of the instances where people posted multiple replies, they reported seeing foxes repeatedly in the same place in summer (June, July and August) (χ^2^ = 25.36; df = 11; p<0.05). Foxes were seen most often at night (49%), but also at dusk (29%) and in daylight (22%). People most often saw foxes in towns while walking (45%) or when using a motor vehicle (27%), and about a quarter of people saw foxes from the window of a building.

### Urban fox behaviour

**Figure 4:**
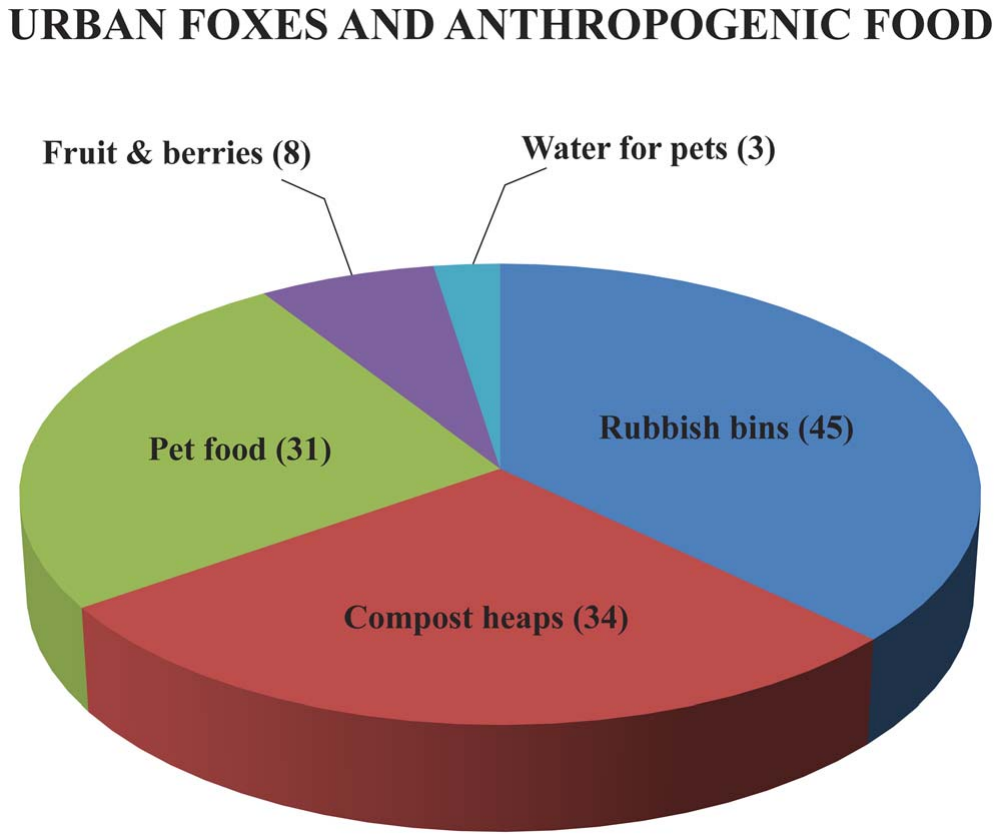
Food types exploited by urban foxes in Estonia (see also Table 1). Numbers indicate the number of people reporting fox activities.

**Figure 5:**
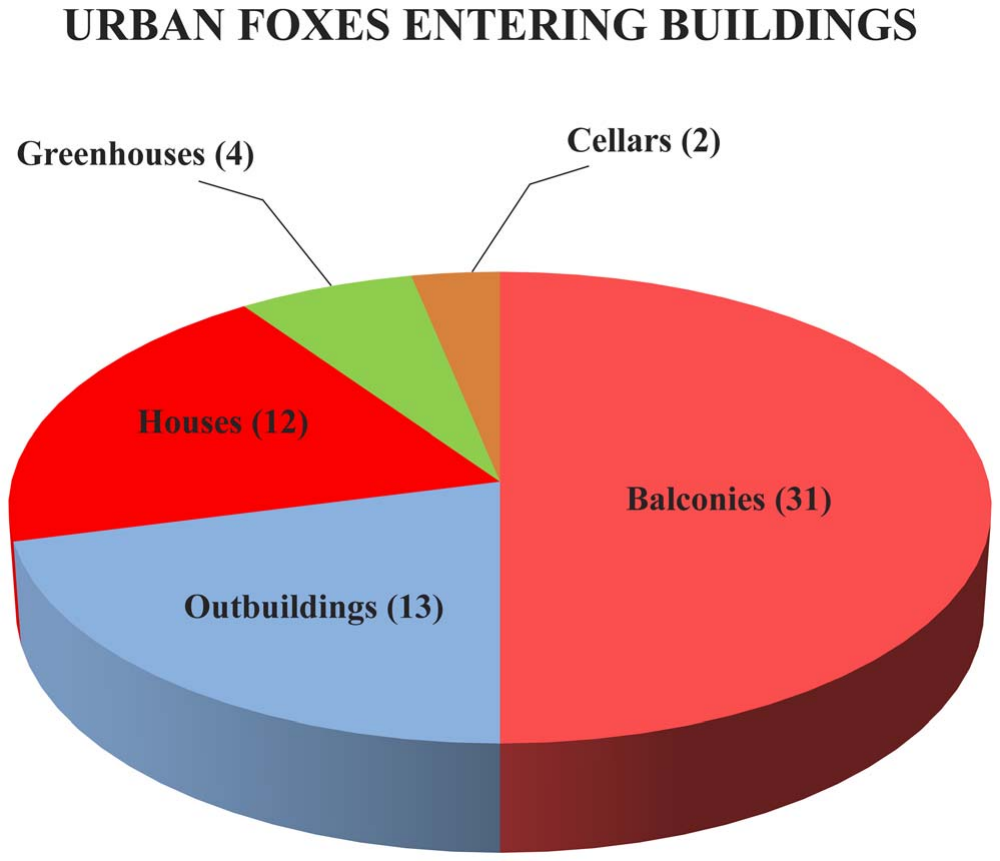
Different buildings exploited by urban foxes in Estonia (see also Table 1). Numbers indicate the number of people reporting fox activities.

Although most reports were of foxes walking or running, people also saw foxes engaged in other activities: some were seen searching for food (Fig. 4; Table 1) in rubbish bins or in compost heaps, some ate food or drank water meant for dogs or cats, and some ate fruit or berries in gardens. Foxes were also seen to disturb domestic animals and enter human buildings (Fig. 5; Table 1) such as balconies or even houses. Other activities were also reported: eating wild birds (4 times), hedgehogs (3), frogs (1) and winter food meant for garden birds (4), and it was sometimes noted that foxes specially watched for traffic and crossed roads at safe moments (5). Foxes were also observed marking their territories (3), vandalizing rubbish bags (4), chasing cats (13) and being chased by cats (4) and crows (2). Sometimes foxes came to gardens where people were barbecuing meat (4) and several times foxes carried away shoes left outside (5). It was reported twice from Tartu that foxes were seen mating. Forty-one responses noted that foxes were surprisingly fearless despite humans or even humans with dogs walking close by, and in only 18 cases it was mentioned that the fox ran away.

**Table 1:** Red fox activities in Estonian urban areas.

Fox activity	Nr of responses	%
Walking or running	1103	83.5
Searching for food in rubbish bins	45	3.4
Disturbing domestic animals	36	2.7
Searching for food in compost heaps	34	2.6
Entering balconies	31	2.3
Eating pet food	31	2.3
Entering outbuildings	13	1.0
Entering houses	12	0.9
Eating fruit and berries in gardens	8	0.6
Entering greenhouses	4	0.3
Drinking water for pets	3	0.2
Entering cellars	2	0.2

In 42% of cases, foxes were seen near the home of the respondent (in their garden, in the street next to their home, etc.). Of these cases, 38% of respondents kept a dog or dogs outside, while 48% did not have a dog (others did not specify). Most of the dogs that were kept outside and could move freely in the garden were large breeds (e.g. German Shepherd, Golden Retriever) or mongrels.

The majority of participants replied that the fox noticed them (58%), while others thought that the fox did not detect them or were unsure (42%). In cases where foxes noticed the observer, about 25% of foxes ran away, about 31% walked away, and 44% did not react or even moved towards the observer. In the great majority of reports, participants saw foxes individually (93%), but sometimes groups of two (4%) or three (0.5%) foxes were also seen together. Foxes were occasionally observed with cubs (3%).

Some participants reported that foxes attacked their domestic animals. In some cases foxes killed and ate domestic animals, whereas in others they killed the animal but did not eat it (Table 2). Foxes killed poultry and cats most often. They attacked cats without killing them 9 times, chickens 3 times and dogs 4 times. Thus, although dogs were sometimes attacked, they were the only species not killed or eaten.

**Table 2:** Red fox attacks on domestic animals in urban areas in Estonia.

Action	Dog	Cat	Poultry	Rabbit
Attacked	4	9	3	0
Killed	0	4	4	1
Killed and eaten	0	5	6	0

To evaluate the availability of anthropogenic food to foxes, participants who saw foxes near their home were asked to specify if there was any accessible anthropogenic food nearby. Less than one fifth of participants (16%) reported that they sometimes left food outside for domestic animals, while 11% reported doing so frequently. Accessible compost heaps with food waste were present in 40% of cases where foxes were observed close to a participant's home.

### Foxes infected with sarcoptic mange

The appearance of foxes was in most instances reported to be normal. However, some people observed foxes with alopecia (partially hairless coats on the body or tail area). Those foxes (8%) were probably infected with sarcoptic mange, which was common in rural areas during the same period. We believe that people mainly reported mangy foxes rather than moulting foxes as mangy foxes lose most of their hair (both down and guard hair) from infected regions, whereas in moulting foxes the fluffy look persists, because the long guard hairs are not shed. Infected foxes were most often seen in spring and summer (χ^2^ = 15.73; df = 3; p = 0.01). The proportion of records reporting foxes with mange was positively related to the size (km^2^) of the urban area (χ^2^ = 4.14; df = 1; p = 0.04; parameter estimate ±s.e = 0.0036±0.0018) and to the human population of the urban area (χ^2^ = 4.94; df = 1; p = 0.03; parameter estimate ±s.e = 1.5e^−6^±6.7e^−7^). Altogether, partially hairless foxes were seen in 13 towns. Most such sightings came from Tartu (45 times), Tallinn (33 times) and Pärnu (6 times) (Fig. 2). Participants also noted in open questions that some foxes were skinny (61 times), limping or injured (9 times).

## Discussion

### Rapid urbanization of foxes throughout the country

Our questionnaire revealed that red foxes have colonized most urban areas in Estonia. Foxes were reported in 33 towns out of 47 (70%), and this can be regarded as a minimum number, as we may simply not have attracted respondents from all towns. It is notable that foxes have colonized not only the small towns and peripheral areas of towns, where the natural environment is close, but also the largest cities in Estonia, such as Tallinn, Tartu and Pärnu. On top of that, they have been sighted most frequently in the central parts of these cities. Foxes were recorded more frequently from the larger and more populated towns. These towns may offer more abundant resources for feeding, but the result may to some extent reflect a reporting bias since the number of potential observers is also larger in more populated towns.

Relatively rapid colonization of urban areas by foxes is most likely an outcome of the successful oral vaccination of wild animals against rabies that started in Estonia in autumn 2005 [21], [30]. Shortly after the vaccination program began, the abundance of foxes increased rapidly, and a proportion of the population was probably forced to search for food and mates outside of the species' usual habitat. By 2008, foxes were already abundant in many Estonian urban areas. A similar pattern has been reported in Switzerland, where foxes colonized urban areas, including the largest cities, shortly after a similar oral vaccination campaign against rabies in the 1980s [10].

### Fox dens in urban areas

Our survey indicates that foxes commonly forage in urban areas; however, in most cases it is unclear whether this reflects permanent occupation of the urban area or rather a temporary (e.g. within-night or seasonal) visit. In some cases at least, it is clear that foxes also live and breed in urban areas. Natal dens were reported in six towns and in some cases cubs were observed near a den. One den in Tartu was less than 1 km from the Town Hall Square, close to a street with relatively heavy traffic. The fact that people observed two or more foxes together, and also with cubs, further supports the idea that foxes also breed in urban areas and are not particularly inhibited by close human proximity [10], [18]. In addition to the eleven dens reported through questionnaires, we know of one further urban den (in Paide). Thus, despite their recent urbanization, Estonian urban foxes already appear to be “true” urban foxes in the sense that they can live and breed in urban areas.

### Behaviour of foxes in urban area

Foxes were most frequently seen at night (49%, 673 times). This is in accordance with previous observations that urban foxes avoid moving during the day and in the presence of active traffic [20]. In rural areas, foxes are often active during the daytime if encountering people is unlikely. Foxes were most often seen in December and January, which represents both the breeding season and the coldest period of the year. Fox movements are greater during the breeding season, and visits to urban areas may therefore become more frequent. Similarly, foxes prefer to move during hours of darkness, so more frequent visits to urban areas during the darkest period of the year might also be for this reason. However, the availability of anthropogenic food items seems likely to be an important reason for foxes to visit and inhabit Estonian urban areas, especially when the availability of natural food resources is reduced in winter (especially when there is deep snow cover, as was the case when the questionnaire was implemented). The proportion of fox records coming from winter (December, January and February) was generally higher in larger towns, though the relationship was marginally non-significant. Such a trend might indicate that larger towns offer more abundant food resources or greater thermodynamic benefits.

Foxes were reported multiple times from the same location most frequently during summer. This may be explained by territoriality: during summer most fox movements occur in territories close to their dens [31], or it may be that foxes use dependable anthropogenic resources to feed their young.

As mentioned above, foxes were not afraid to visit or even live close to the city centre, and they were seen most often in the central part of the three largest cities in Estonia. However, since people may be most active in the central parts of the cities both during the day and at night, it is possible that this reflects reporting bias. Other studies have shown that foxes prefer less disturbed residential districts and suburbs for diurnal shelter and natal dens and make short visits to city centres to access food resources [18], [32].

Our survey provided evidence of the type of flexible and bold behaviour that presumably facilitates adaptation to new environments [7]. Although road-killed foxes were occasionally reported and there are more cases known in other urban areas of Estonia (Authors' observation), foxes were reported to cross roads primarily when traffic subsided. Nevertheless, traffic accidents are generally the main cause of death for urban foxes [20]. When encountering people, foxes often walked away slowly, ignored the observer or even approached the observer. Wild mammals in their natural environment usually avoid encountering people. In several cases, foxes were unafraid of humans walking with dogs on a leash and some foxes took food directly from people. This bold behaviour can also explain why some residents were afraid of foxes, though direct attacks on humans remain extremely rare [33] and none were reported in Estonia.

### Conflict with human populations: killing domestic animals and potential spillover of dangerous zoonotic pathogens

Foxes attacked, killed and ate domestic animals, mostly poultry and cats. Foxes were also seen to chase cats. While dogs were occasionally attacked, they were never reported to be killed or eaten. Fox attacks cause conflicts with humans, especially in smaller towns where domestic animals are kept more loosely and such incidents may therefore be more frequent. Foxes were reported as a considerable threat to livestock and poultry in periurban areas in preindustrial and early industrial Finland [34]. Remnants of cats and dogs have also been found in urban fox diet studies [16], [35]. Therefore it is possible that urban foxes predate on domestic pets, but the results of this study suggest that the rate of such incidents is low.

Since foxes are now common in many Estonian towns, the risk of pathogen spillover is increased. In Estonia, foxes have been identified as a vector for severe infectious diseases including rabies, sarcoptic mange and alveolar echinococcosis. Rabies is now virtually eradicated from Estonia following a successful anti-rabies vaccination campaign [30]. However, in recent years not only has the fox population started to increase, but so has the number of animals infected with sarcoptic mange, a highly contagious zoonosis caused by the burrowing mite *S. scabiei*. The parasite also infects domestic mammals, mostly dogs. The disease causes considerable nutritional stress and compromises the immune system of infected animals [14]. About 8% of foxes seen in towns were partially hairless and therefore most likely infected with sarcoptic mange, representing a potential source of infection for domestic animals. The proportion of records reporting a mange-infected fox was higher in larger towns. Infected foxes may need more energy, and larger towns may offer more anthropogenic food, a warmer environment and more opportunities for shelter.

Approximately 30% of rural red foxes in Estonia are infected with the fox tapeworm *E. multilocularis* which causes alveolar echinococcosis, a life-threatening disease to humans [36]. Moreover, the parasite was very recently detected in Estonian urban foxes [37] and cases of human echinococcosis are rising [38]. Another dangerous parasite in the same genus, *E. granulosus* [39], has also been found in Estonia [40], [41]. This zoonotic pathogen is spread in urban areas primarily by dogs and it has been recently detected in dog scats found in an urban area in Estonia (Laurimaa et al., unpublished results). Thus, it seems likely that the risk to humans of parasite infection has risen considerably as foxes have started to visit urban areas. Monitoring parasite spillover into urban areas is consequently a high priority.

Given these disease risks, the general public must be informed of how to avoid parasite transmission without promoting an unsubstantiated fear of foxes. Anthropogenic food has been shown to be a major reason why foxes visit urban areas (e.g. [16]). The same probably applies to urban foxes in Estonia since accessible rubbish bins and compost heaps, as well as food destined for domestic animals, were reported to be readily available and used by urban foxes. Even 10% of red foxes from Estonian rural areas had consumed some anthropogenic food (Soe et al., unpublished results) and similar results have been shown in Alpine red foxes [42]. Reducing access to food items would appear to represent the most effective means of reducing the number of urban foxes.

## Supporting Information

S1 Figure **Distribution of survey respondents according to age.**
(TIFF)

S1 Information **Web-based questionnaire: red foxes in urban areas in Estonia.**
(DOCX)

S2 Information **Web-based questionnaire: raw data spreadsheet.**
(XLSX)
