# Supplementary material for: Rapid Urbanization of Red Foxes in Estonia: Distribution, Behaviour, Attacks on Domestic Animals, and Health-Risks Related to Zoonotic Diseases
Source: PLoS One. 2014 Dec 22;9(12):e115124. doi: 10.1371/journal.pone.0115124 (PMC4274007; doi:10.1371/journal.pone.0115124)
Supplement: S1 Information — Web-based questionnaire: red foxes in urban areas in Estonia. (DOCX) [file pone.0115124.s002.docx]

**Information S1. Web-based questionnaire: red foxes in urban areas in Estonia**

**1. I am answering the questionnaire:** for the first time/second time/third time/more than third time/

*Please choose one answer*

**2. Your sex:** male/ female/

*Please choose one answer*

**3. Your age:** <10 years/ 11-20/ 21-30/ 31-40/ 41-50/ 51-60/ 61-70/ >70/

*Please choose one answer*

**4. In which town did you see the fox?**

*Please choose the town from the list (all 47 towns in Estonia were provided)*

**5. In which part of the town did you see the fox?**

*Please write the name of the part of the town. If you do not know, write „do not know“*

**6. Nearest address**

*Please write the address of the building closest to where you saw the fox. If you do not know the address, then please describe the location (street, nearest church, store, etc)*

**7. In which year and month did you see the fox in town?**

*Please choose the year and the month, or if you do not remember then choose „do not remember“*

**8. Have you repeatedly seen a fox in the same place:** yes/no/

*Please choose one answer*

**9. If you have repeatedly seen foxes at the same location then describe the time period**

*For example: 2010, November-December*

**10. At what time of day did you see the fox:** at night/at dusk/during daylight hours

*Please choose one answer, but if you have seen a fox repeatedly then choose more than one answer if necessary.*

**11. Did you see the fox:** from inside a building/from a vehicle/while riding a bike/while walking

*Please choose one answer, but if you have seen a fox repeatedly then choose more than one answer if necessary.*

**12. The appearance of the fox was:** usual (did not notice anything special)/ the body or tail area was partially hairless

*Please choose one answer; if you wish to add something you can do so in the next section.*

**13. Provide additional information about the appearance of the fox (optional)**

**14. Fox behaviour:** ate pet food/drank water for pets/ate fruit and berries from a garden/ rummaged through rubbish bins/ate food waste/disturbed domestic animals/entered the house/entered balcony/entered greenhouse/entered cellar/entered outbuildings/did not do anything special

*Please choose as many answers as needed. If you wish to add something you can do so in the next section.*

**15. Provide additional information about the fox behaviour (optional)**

**16. Did the fox notice you:** yes/no/do not know

*Please choose one answer*

**17. If the fox noticed you then the fox:** was not disturbed at all/moved towards you/left slowly/ran away/ran away when you stopped the vehicle

*Please choose one answer*

**18. Was the fox alone:** alone/together with another adult fox/together with two adult foxes/together with three or more adult foxes/with cubs

*Please choose one answer*

*Please answer the next eight questions if you have domestic animals and you observed the fox near your home!*

**19. Do you keep one or more dogs outside:** one dog/two dogs/more than two dogs/no dogs

*Please choose one answer*

**20. Is the dog(s) leashed or can it move freely:** leashed at all times/leashed only during the daytime/leashed only at night/can move freely in a fenced garden/can move freely outside the garden

*Please choose multiple answers if necessary*

**21. If you have a dog(s) outside, please describe their age, breed and weight**

*Please write „mongrel“ for mixed or uncertain breeds*

**22. Have you or a family member observed a fox doing the following to your domestic animal:** attacking/killing/killing and eating/none of these

*Please choose one answer*

**23. If the fox has attacked, killed or eaten domestic animals, then it did it do this:** once/repeatedly

*Please choose one answer*

**24. Which domestic animal species was/were attacked, killed or eaten by the fox**

*For example: attacked dog once, killed cats twice*

**25. Do you leave food outside for domestic animals:** no/sometimes/regularly

*Please choose one answer*

**26. Are there any accessible refuse heaps with food waste near your home:** no/sometimes/always

*Please choose one answer*

*Thank you for completing the survey!*
